# Anaerobic Fermentation of Poly(3-hydroxybutyrate-*co*-3-hydroxyvalerate) Plasticized with Glycerol Trilevulinate into Volatile Fatty Acids

**DOI:** 10.1021/acsomega.6c00869

**Published:** 2026-03-31

**Authors:** Elena Togliatti, Yong Jin, Luca Lenzi, Davide Morselli, Micaela Degli Esposti, Paola Fabbri, Daniel Milanese, David P. B. T. B. Strik, Corrado Sciancalepore

**Affiliations:** a Department of Systems and Industrial Technologies Engineering, 9370 Università di Parma, Parco Area delle Scienze 181/A, Parma 43124, Italy; b National Interuniversity Consortium of Materials Science and Technology (INSTM), Via G. Giusti 9, Firenze 50121, Italy; c Environmental Technology, 4508 Wageningen University & Research, Wageningen 6708 WG, The Netherlands; d Department of Civil, Chemical, Environmental and Materials Engineering, 9296 Università di Bologna, Via Terracini 28, Bologna 40131, Italy

## Abstract

Polyhydroxyalkanoates (PHAs) are a promising class of biobased and biodegradable polymers; however, their inherent brittleness and limited processability hinder widespread application. The glycerol trilevulinate (GT) bioplasticizer has been proposed to enhance the mechanical properties of PHAs without compromising their biodegradability. This study investigates the effect of GT at different concentrations on the anaerobic fermentation of poly­(3-hydroxybutyrate-*co*-3-hydroxyvalerate) (PHBV) to volatile fatty acids (VFA), focusing on microbial conversion efficiency. These VFA are platform chemicals serving various applications, including resynthesis of PHAs. PHBV/GT blends were characterized in terms of their thermal and morphological properties. The results show that the increased GT percentage reduces the crystallinity and melting temperature, thereby enhancing polymer flexibility. Anaerobic fermentation experiments were conducted using (i) prehydrolyzed PHBV/GT fermentation at fixed 10 wt % GT content and (ii) direct fermentation of PHBV/GT solid particles at different GT concentrations. The findings show that GT-plasticized PHBV was successfully converted into VFA, with acetate and *n*-butyrate being the predominant fermentation products. While low concentrations of hydrolysate (4–10 g SCOD/L) were efficiently converted, higher concentrations (20 g SCOD/L) did not lead to fermentation, suggesting potential microbial toxicity. The different contents of GT did not affect the microbial conversion process. These findings support the use of GT as plasticizer for PHA-based materials and development of renewable, biodegradable, and microbial recyclable plastic products.

## Graphical abstract

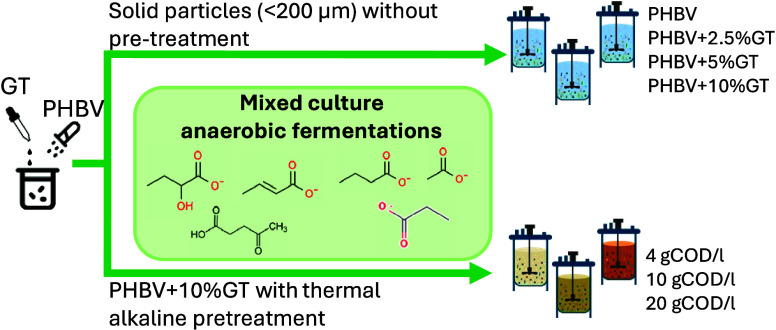

## 1 Introduction

Polyhydroxyalkanoates (PHAs) represent a promising biodegradable, biocompatible, and biobased class of polymers that are considered potential alternatives to traditional plastics.^1^ These polymers can be synthesized by microorganisms wherein the excess of carbon source is converted into intracellular storage granules.^2,3^ Furthermore, PHAs can be produced from various feedstocks, including sugars, vegetable oils, wastewater, and food waste.^4–8^ The circular nature of PHAs, which is evidenced by the use of renewable biomass and biowaste-derived materials as feedstocks,^9^ makes them particularly attractive in the context of global efforts to reduce reliance on fossil-based resources.^10^ The versatility of PHAs stems from their chemical structure, which varies in terms of chain length and comonomer composition. These variations allow for a wide spectrum of physical, thermal, and mechanical properties, enabling PHAs to be tailored for specific applications such as supporting tissue regeneration in the biomedical field or for use in the packaging industry, among others.^6,11–13^ PHA-based products can still maintain a certain level of biodegradability, which could help mitigate plastic pollution from wear during use or (unintentional) spillage. Based on their chain length, PHAs are classified into three categories: short-chain-length PHAs (scl-PHAs), medium-chain-length PHAs (mcl-PHAs), and long-chain-length PHAs (lcl-PHAs).^14^ Scl-PHAs, such as poly­(3-hydroxybutyrate) (PHB) and poly­(3-hydroxybutyrate-*co*-3-hydroxyvalerate) (PHBV), are among the most extensively studied members of the polymer class.^15,16^ PHBV, in particular, has been recognized for its improved flexibility and reduced brittleness compared to PHB, with its properties largely determined by the 3-hydroxyvalerate (3-HV) content.^15^ Thanks to this wide range of properties and especially their biodegradability, PHAs have been attracting an increased interest over the past decade. The PHAs' market share is expected to further grow from 102 kilotons in 2024 to 975 kilotons in 2029.^17^ Still, certain PHAs face challenging brittleness, given the high degree of crystallinity ranging between 50 and 70%, which^18^ limits (thermal) processability and thus adoption of PHAs.^19,20^

To address this limitation, there is a growing interest in incorporating biobased plasticizers, which have demonstrated the capacity to enhance the flexibility, toughness, and processability of PHAs.^21,22^ The use of biobased and biodegradable additives in new PHAs raw materials—and in the products made from them—is important for maintaining their renewability and biodegradability benefits.^15,22,23^ A range of additives have been examined in this context, with particular attention being paid to plasticizers obtained from levulinic acid,^24^ and glycerol-based plasticizers.^25^ Notably, glycerol trilevulinate (GT) bioplasticizer is produced through the esterification of levulinic acid and glycerol, which are derived from renewable resources, thereby providing a biobased alternative to conventional plasticizers, such as acetyl tributyl citrate (ATBC), tributyl citrate (TBC), triethyl citrate (TEC), and glycerol triacetate (GTA).^21,26,27^ Preliminary research has demonstrated that GT significantly enhances the mechanical properties of PHAs while preserving their biodegradability. The high compatibility of GT and PHAs also leads to a fine-tuning of the polymer’s crystallinity and melting temperature, thus enhancing workability and potentially broadening the range of applications for PHAs-based materials.^28,29^ Furthermore, it was demonstrated that GT is capable also to enhance the enzymatic hydrolysis of semicrystalline biodegradable polyesters such as PLA.^30^

In addition to material optimization, the development of alternative end-of-life management strategies for PHA-based products is crucial for realizing their potential within circular bioeconomy frameworks.^31^ Nowadays, PHAs are used in food services/packaging (like cups, pads, trays, and straws) and in agriculture (in mulching films and plant clips), but also consumer goods like clothing may become available soon.^17,32–36^ The inherent biodegradability of PHA polymers enables their use in applications where this property is essential, such as mulching films. In other applications, such as coffee pods, it may be advantageous for the end-of-life product to decompose under controlled conditions, allowing the recovery of bioresidues for compost production under aerobic conditions or for biogas generation through anaerobic digestion.^37,38^ However, for these and other PHA-based products, also new recycling routes can be developed that do not degrade the products but rather recover the waste materials to make new products. Recently, alternative anaerobic fermentation processes have garnered significant attention as a microbial recycling strategy for converting PHAs and other biodegradable products, into valuable volatile fatty acids (VFA).^39–43^ These VFA, including acetate and butyrate, can serve as precursors for chemicals, fuels, and even new scl-PHAs, thereby partly closing the material loop and supporting a circular production model.^44,45^

Previous PHAs fermentation into VFA research was done with milled or prehydrolyzed commercially available PHAs pellets.^41–43^ One study explored hereby the effects of pretreatment strategies, such as neutral, acid, or alkaline hydrolysis, on the degradation efficiency of PHAs.^40^ Alkaline conditions have been shown to enhance hydrolysis, facilitating microbial access to the polymer matrix and accelerating the conversion of PHAs into VFA.

Despite the growing interest in biobased plasticizers, the majority of current studies concentrate on conventional, non-biobased plasticizers, which are demonstrated to hinder microbial metabolism during anaerobic digestion.^46^ However, research on the anaerobic fermentation of bioplasticizers remains limited. Moreover, anaerobic fermentation is frequently employed with the objective of biogas methane production as opposed to VFA production.^38,47^ To date, no studies have examined how adding plasticizers to PHAs influences their fermentation into VFA.

Therefore, the objective of this study is to investigate the biodegradation of PHBV/GT blends, with a specific focus on understanding how the bioplasticizer concentration affects bioconversion and the formation of fermentation products under anaerobic conditions. Specifically, two experimental approaches were employed: (1) the fermentation of alkaline-hydrolyzed PHBV/GT blends to examine subsequent microbial conversion at different supplied concentrations and (2) the fermentation of solid PHBV/GT particles with different GT percentages to determine whether it would affect bioconversion.

To describe the effect of GT on the polymer, the thermal and morphological properties of PHBV/GT blends were characterized. Thermal analysis provided insights into the influence of GT on the melting, crystallization, and glass transition temperatures and crystallinity degree of PHBV. In the fermentation experiments, the production of VFA was monitored to evaluate the possibility of microbial conversion and the impact of GT concentration on microbial metabolism. The results show that using GT as bioplasticizer does improve material properties and does not compromise the fermentability of the material into VFA.

This study provides, to the best of the authors’ knowledge, the first evaluation of PHBV/GT blends in anaerobic fermentation and clarifies how GT content influences microbial degradability and VFA production. The findings support the development of improved PHA-based materials by addressing challenges in bioplastic manufacturing, as well as end-of-life strategies.

## 2 Materials and Methods

PHBV pellets were purchased from Gruppo MAIP (IamNature B6 T P001Y) with a molecular weight Mn of 240,000 Da and a nominal 3-HV content of 3% mol, as declared by the data sheet. The bioplasticizer GT was synthesized as previously reported^28^ and used without any further processing in different concentrations.

PHBV/GT blends were produced according to a methodology previously followed in ref 29. Briefly, the PHBV granules were roughly ground (IKA A11 basic) under cryogenic conditions with liquid N_2_, and then the polymer matrix was mechanically mixed with the GT in different concentrations by weight, denominated as listed in Table 1.

**Table 1:** Characteristics of PHBV and Various GT Contents

**sample code**	**polymer content** (wt %)	**plasticizer content** (wt %)
PHBV	100	0
PHBV2.5GT	97.5	2.5
PHBV5GT	95	5
PHBV10GT	90	10

After extrusion, which took place in a twin-screw extruder (RES-2 P/12A Explorer Extruder, Zamac Mercator, Poland, conditions are described in Table S1), grinding (IKA A11 basic) and sieving (stainless steel sieve from Retsch, Germany) were performed to obtain a powder with a particle dimension below 200 μm to ensure a higher surface for the microbial fermentation.

The nutrient medium was prepared with the following composition: 0.1 g/L yeast extract (Sigma-Aldrich), 20 mL/L stock solution I and 20 mL/L stock solution II, 1 mL/L vitamins, and 0.5 mL/L trace metals (see Table S2). In addition, 5 g/L of sodium 2-bromoethanesulfanoate (BES, 98%, Merck) was added to the medium to inhibit methane production in favor of VFA. The chemicals used for the fermentation medium followed a previously established protocol.^40^ The fermentation inoculum (5% v/v) consisted of an undefined open culture composed of cow rumen and chain elongation consortia derived from previous experiments,^48^ including lactate-, ethanol-, and glucose-based fermentation broths. The inoculum was prepared with ∼57% cow rumen and ∼14% each of ethanol-, lactate-, and glucose-based fermentation effluent. Before use, it was flushed with N_2_ for 10 min and stored at 4 °C. The inoculum pH was measured at 7.62.

### 2.1 Material Characterization

#### 2.1.1 Morphological Analysis

##### 2.1.1.1 Granulometry Characterization

Size distribution curves and particle dimensions of the powder, expressed by the mean diameter and the D10, D50, and D90 standard percentiles, were obtained with a Mastersizer 3000 laser granulometer (Malvern Instruments Ltd., Malvern, UK) in dry mode and a Fraunhofer approximation. This method applies when the optical properties of the material are unknown. This is the case here because the particles are larger than 20 μm and opaque, which produces minimal light scattering at small angles. D10, D50, and D90 represent the diameters below which 10, 50, and 90% of the particles fall, expressed by volume density.

##### 2.1.1.2 Microscopy Investigation

To observe the microscopical surface aspect of the plastic particles, a scanning electron microscope (SEM) was utilized. Field emission gun SEM (FEG-SEM, Nova NanoSEM 450, FEI Company, USA) images were acquired by making use of the backscatter detector. All images were acquired using an accelerating voltage of 15 kV, an arbitrary spot size of 4, and a working distance of approximately 6 mm.

Additionally, an optical microscope (Nikon, SMZ800) was used to observe the morphology of the plastic particles before and after the fermentation process to compare any changes in the morphology of the particles due to the fermentation process.

#### 2.1.2 Thermal Properties

##### 2.1.2.1 DSC

The thermal properties of the formulations were investigated by differential scanning calorimetry (DSC). Approximately 13.5 mg of each material was analyzed in duplicates with a Q200 instrument (TA Instruments, USA) under an inert N_2_ atmosphere with a purge flow of 50 mL/min. The samples were first equilibrated at a temperature of −60 °C for 5 min, and then a heating ramp of 10 °C/min was applied to reach 200 °C, followed by a cooling cycle to −60 °C and a second heating cycle to 200 °C.

The curves obtained from the DSC were then processed by using TA Universal Analysis 2000 software (TA Instruments). The melting temperature (*T*_m_) and the glass transition temperature (*T*_g_) were calculated, respectively, as the temperatures corresponding to the peak of the phase transition and the temperature corresponding to an inflection from the second heating scan. The crystallization temperature (*T*_c_) was obtained from the cooling scan.

The enthalpies of melting (Δ*H*_m_) and crystallization (Δ*H*_c_) were calculated as the area of the thermogram peaks of the related transitions. The degree of crystallinity (χ_c_) was calculated as in eq 1, where Δ*H* ^0^_m_ = 146 J g^–1^ ^49^ is the standard melting enthalpy of fully crystalline PHBV and *W*_pol_ is the mass fraction of the polymer, calculated as the polymer wt % from Table 1 divided by 100.

$$\chi_{c}=\Delta H_{m}/(\Delta H_{m}^{0}\times W_{\mathrm{pol}})\times 100$$ 1

##### 2.1.2.2 TGA

The thermal stability of PHBV at different plasticizer concentrations was analyzed using thermal gravimetric analysis (TGA), performed through a PerkinElmer TGA 8000. Approximately 6 mg of the samples was placed in a platinum crucible and subjected to a temperature ramp of 10 °C/min from 30 to 600 °C under an inert N_2_ atmosphere at atmospheric pressure, with a 20 mL/min purge flow. Measurements were conducted in duplicate.

The decomposition temperature (*T*_d_) was calculated as the inflection point of the curve representing the relative mass of the sample (*m*_r_ %) as a function of temperature and the onset temperature (*T*_onset_) as the point at which the tangent to the initial baseline intersects with the tangent from the steepest part of the degradation curve. *T*_onset_ marks the beginning of a significant weight loss. *m*_r_ (eq 2) is calculated to normalize each curve as the ratio of the mass of the sample during the measurement (*m*_t_) and its initial mass (*m*_i_):

$$m_{r}=m_{t}/m_{i}\times 100$$ 2

The results are presented as the percentage change in sample mass as a function of the temperature.

### 2.2 Experimental Design for Anaerobic Fermentation

Two series of comparative experiments, described in Table 2, were designed in this study: (I) open-culture fermentation of PHBV hydrolysates where PHBV10GT was initially pretreated via hydrothermal alkaline hydrolysis; (II) open-culture fermentation of PHBV solid particles with various percentages of GT.

**Table 2:** Overview of the Experimental Design and Conditions of the Performed Fermentations

**no.**	**experiments**	**substrate**	**concentration** ^a^	**duration**	**initial pH** ^b^
I	hydrolysate fermentation (HF)	PHBV10GT hydrolysates	4 g SCOD/L	15 days	6.8 ± 0.1
			10 g SCOD/L	15 days	6.7 ± 0.0
			20 g SCOD/L	15 days	6.8 ± 0.0
II	solid fermentation (SF)	PHBV	50 g_plastic_/L	120 days	6.8 ± 0.1
		PHBV2.5GT	50 g_plastic_/L	120 days	6.8 ± 0.0
		PHBV5GT	50 g_plastic_/L	120 days	6.8 ± 0.1
		PHBV10GT	50 g_plastic_/L	120 days	6.7 ± 0.0

a SCOD stands for soluble chemical oxygen demand.

b The initial pH values are measured after bottle preparation.

#### 2.2.1 Hydrothermal Pretreatment (HTP)

The hydrolysis of PHBV pellets was conducted in an 800 mL benchtop stirred reactor (Parr 4857, Parr Instruments Moline, Illinois, USA). 15 g of PHBV10GT was dispersed in 700 mL of an aqueous solution of NaOH 0.25 M. The obtained suspension was heated up to 160 °C within an hour, and the temperature was maintained for the following 22 h. Subsequently, the temperature was cooled to room temperature by the cooling solvent (Thermal H10). A continuous stirring, at 85 rpm, was continued for the entire HTP. No pressure control measures were used; instead, monitoring of the overpressure was conducted. The graph reporting the temperature and the pressure profiles during the process is shown in Figure S1. After the pretreatment, the obtained hydrolysates were collected by centrifugation at 6920 rpm for 18 min (Hermle, Z36HK) and filtered on a membrane having porosities of 0.45 μm (CHROMAFIL Xtra, Macherey-Nagel, Düren, Germany). The final pH was 11.4.

#### 2.2.2 Open-Culture Fermentation of PHBV Hydrolysates and of Solid Particles

For the first set of experiments, PHBV10GT was employed. The obtained soluble chemical oxygen demand (SCOD) of hydrolysates, measured with LCK514 kits (HACH GmbH, Germany) after filtration by 0.45 μm membrane and an appropriate dilution of the filtrate, was 35.39 g SCOD/L derived from 13.5 g of PHBV and 1.5 g of GT. This hydrolysate was diluted to 4, 10, and 20 g SCOD/L with demineralized water. 250 mL glass serum flask bottles were used as batch reactors. For hydrolysate fermentation (HF), the working volume was 47.5 mL, achieved by mixing the diluted hydrolysates and nutrient medium, prepared with composition described in Table S2. Samples of the control groups were prepared analogously but using demineralized water instead of hydrolysates. After the preparation of the liquid medium, the resulting pH was found to be acidified to approximately 5.8; therefore, the pH was adjusted to 7.0 ± 0.1 with 4 M KOH. The bottles were then sealed with a rubber septum and N_2_ was flushed for 10 min to ensure anaerobic conditions; subsequently, 2.5 mL of the open-culture inoculum was injected, to bring the total working volume to 50 mL, and finally, the headspace was filled with an N_2_/CO_2_ (80/20 vol %) atmosphere at 1.5 bar with a gas exchanger (SC920G, KNF Neuberger, Freiburg, Germany). The sampling occurred at time 0 (*t*_0_) and once every 3 days up to 15 days by collecting 2 mL (stored at −20 °C until the analysis).

For the second set of experiments, which involved solid fermentation (SF), the ground particles of PHBV and PHBV/GT particles with three GT concentrations (2.5, 5, and 10 wt %) were used as a substrate for fermentation without pretreatment. Similarly to the HF, 45 mL of nutrients and demineralized water, 2.5 mL of inoculum, and 2.5 g of plastic particles (plastic density was assumed to 1 g/mL; therefore, 2.5 g was considered as approximately 2.5 mL) were mixed to obtain the working volume. Homogenization of the suspension was achieved with mechanical stirring. As a control reference, reactors with 47.5 mL of demineralized water and nutrients and 2.5 mL of inoculum were prepared. pH adjustment, gas exchange, and inoculation were performed analogously as previously described. For SF experiments, sampling occurred at *t*_0_ and every 30 days for 120 days. Samples of 2 mL volume were collected and stored at −20 °C until the analysis.

For both sets of experiments, all samples were prepared in triplicate, and the fermentation process took place in a temperature-controlled shaker (Innova 44) at 35 °C and 120 rpm.

### 2.3 Analytical Measurements

Before each sampling, the headspace pressure in each batch bottle was measured with a pressure meter (GMH 3151, GHM Group, Greisinger, Regenstauf, Germany) to ensure that overpressure in the headspace atmosphere was maintained, and pH was measured with a pH meter (PHM210, Radiometer Analytical SAS, France).

The headspace gases (N_2_ and CO_2_) were determined by a gas chromatography (GC) system (Shimadzu GC-2010, Kyoto, Japan) equipped with the column in parallel, with a combination of PoraBOND Q (50 m × 0.53 mm × 10 μm; Varian; Part no. CP7355, Agilent, Amstelveen, The Netherlands) and Molsieve 5A (25 m × 0.53 mm × 50 μm; Varian; Part no. CP7538, Agilent, Amstelveen, The Netherlands). A volume of 50 μL was injected at a temperature of 120 °C, whereas the temperatures in the oven and detector were 45 and 150 °C, respectively. The carrier gas consisted of H_2_ at a pressure of 0.6 bar. H_2_ and CH_4_ were detected by another GC system (HP-5890, Hewlett-Packard, Agilent, Santa Clara, California, USA) equipped with an HP Molsieve 5A (30 m × 0.53 mm × 25 μm) column. Argon was used as a carrier gas, and the injection volume was 100 μL. The oven temperature was 40 °C.^50^

The VFA production was analyzed by liquid gas chromatography (GC, Agilent 7890B, Agilent, Santa Clara, California, USA) equipped with an HP-FFAP column (25 m × 0.32 mm × 0.50 μm). The flame-ionized detector (FID) and injection temperatures were 240 and 250 °C, respectively.^51^ Before injection, the samples were centrifuged at 15,000 rpm for 10 min (Eppendorf, 5425), filtered over a 0.45 μm membrane (CHROMAFIL Xtra, Macherey-Nagel, Düren, Germany), properly diluted, and finally acidified with formic acid at a concentration of 15 wt %. The injection volume was 1 μL, and the carrier gas was N_2_, with 1.25 mL/min for the first 3 min and 2 mL/min until the end of the run.^52^ The oven temperature profile was as follows: starting at 60 °C for 3 min, increasing at a rate of 21 °C/min until reaching 140 °C, and then increasing at 8 °C/min until 150 °C, and holding steady for 1.5 min. It then increased at 120 °C/min to 200 °C, maintaining that temperature for 1.25 min. Finally, it increased again at 120 °C/min to 240 °C, where it remained constant for 3 min.

The data obtained from chromatography were analyzed using Chromeleon software (version 7, Thermo Fisher Scientific, Waltham, Massachusetts, USA). The SCOD coming from the VFA identified through the GC was calculated by multiplying the concentration in mg/L obtained from the GC measurement by the appropriate dilution factor, obtaining this way the real concentration and subsequently the chemical oxygen demand (COD) (g COD/g) of each VFA component. As 3-hydroxybutyrate (3HB) and crotonic acid (CA) were only qualitatively determined, due to limitations of the analytical method followed, the denomination CA-3HB will be used to stand for these PHBV hydrolysates. Since CA-3HB is a mixture with an unknown composition, its contribution to SCOD was estimated using the SCOD value of crotonate as a benchmark.

For the hydrolysate fermentation, the unknown SCOD was calculated as the difference between the fed SCOD in the reactors at time 0 (4 g SCOD/L, 10 g COD/L, and 20 g SCOD/L) and the SCOD corresponding to the total VFA identified through the GC at the time of each sampling.

## 3 Results and Discussion

### 3.1 Characterization of Bioplastic Particles

#### 3.1.1 Morphology

Plastic particles were analyzed by SEM (Figure 1a). Upon observation of the particle morphology, it becomes evident that the mechanical grinding of PHBV granules produced irregularly shaped particles. Additionally, the materials are homogeneous at varying plasticizer concentrations. The absence of phase separation or regions where the plasticizer exudes indicates a thorough mixing of the additive within the polymer matrix, suggesting good compatibility between the two components at the studied concentrations.^23^

**Figure 1:**
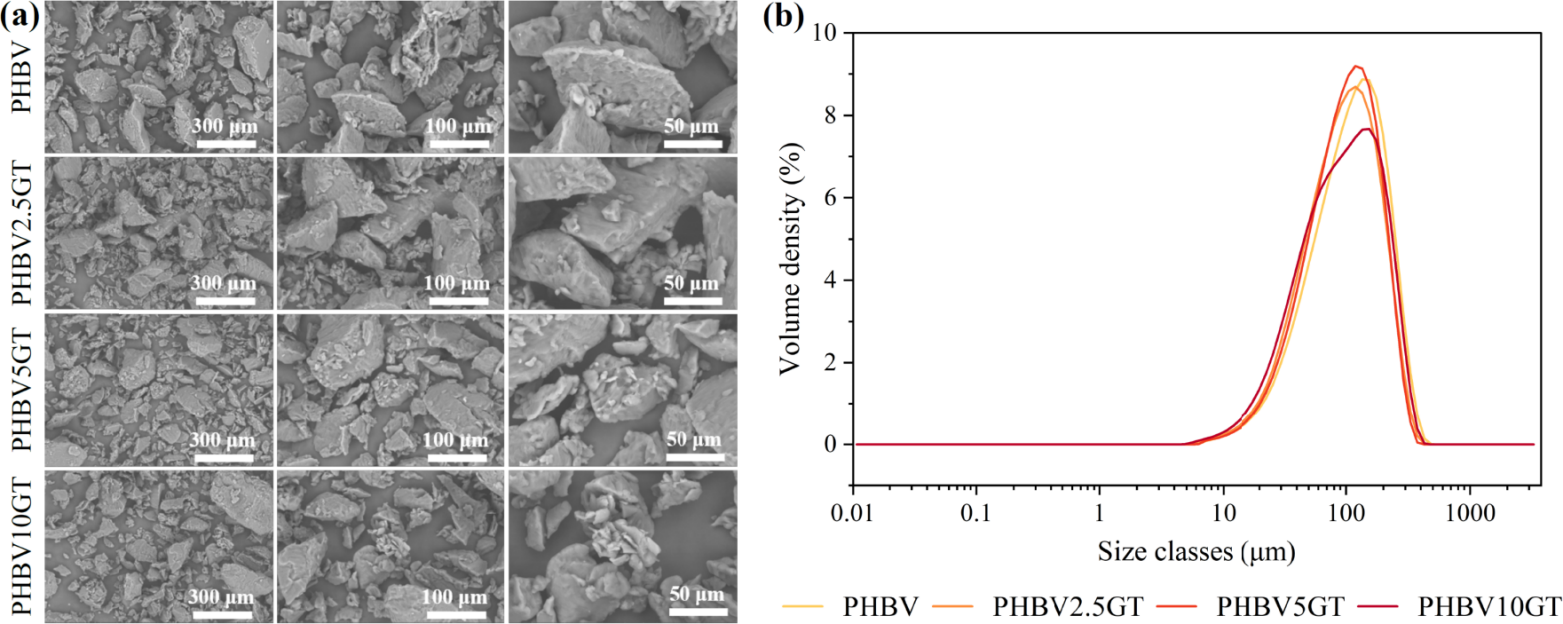
(a) SEM images at magnification of 400× (left column), 600× (middle column), and 1600× (right column). (b) Size distribution curves (mean of two measurements) of the milled materials.

The size distribution curves (Figure 1b) show a consistent trend across all formulations, except PHBV10GT, which exhibits a main peak of lower intensity with a marked shoulder at smaller size class values. Particle size analysis (data reported in Table 3) confirmed the particle size after sieving, with D90 ranging from 203 to 232 μm and a volumetric mean diameter (D4:3) of approximately 110 μm. In contrast, considering the average diameter calculated on the number of particles (D1:0), values approximately 10 times lower than this are obtained. This indicates that most of the particles have a small diameter, allowing them to have a higher specific surface area. The distribution of particle dimensions, and, in particular, the large specific surface area combined with the rough surface texture can be considered a favorable factor for biodegradation, since it is well known that biodegradation depends on the extent of surface area exposed to enzymatic hydrolysis.^53^

**Table 3:** Particle Size Analysis of the Milled Materials

**parameters**	**D10 (μm)**	**D50 (μm)**	**D90 (μm)**	**D**4:3 **(μm)**	**D**1:0 **(μm)**	**specific surface area** (m^2^/kg)
PHBV	38.2 ± 0.1	110.0 ± 1.1	232 ± 0.9	124.0 ± 0.1	19.7 ± 0.3	79.6 ± 0.2
PHBV2.5GT	34.3 ± 0.1	95.1 ± 0.9	205.0 ± 2.1	109.0 ± 1.1	17.9 ± 0.2	89.9 ± 0.6
PHBV5GT	36.3 ± 0.1	98.6 ± 0.6	203 ± 1.7	110.0 ± 1.7	20.1 ± 0.1	85.7 ± 0.1
PHBV10GT	31.2 ± 0.3	95.3 ± 0.5	221.0 ± 0.2	112.5 ± 0.2	16.3 ± 0.9	94.4 ± 0.5

#### 3.1.2 Thermal Properties

##### 3.1.2.1 DSC

In addition to the chemical properties of the polymer, such as molecular structure and molecular weight, the physical properties, in particular, the thermal properties, investigated with DSC, can significantly impact the biodegradation rate.^54^ The full DSC scans are listed in Figure S2.

The amorphous fraction of a polymer experiences the transition from a glassy state to a rubbery state when heated above its *T*_g_. Referring to Figure 2a, *T*_g_ values decrease, as expected, by increasing the amount of plasticizer added to the PHBV. Graphically, the inflection point relative to the transition is more pronounced for the bare polymer, whereas as the amount of plasticizer increases, the inflection flattens and widens. This is due to the intercalation of plasticizer molecules between the polymer macromolecules, thereby increasing the free volume and consequently the molecular mobility at lower temperatures. This mechanism is referred to as “plasticization”.^55^

**Figure 2:**
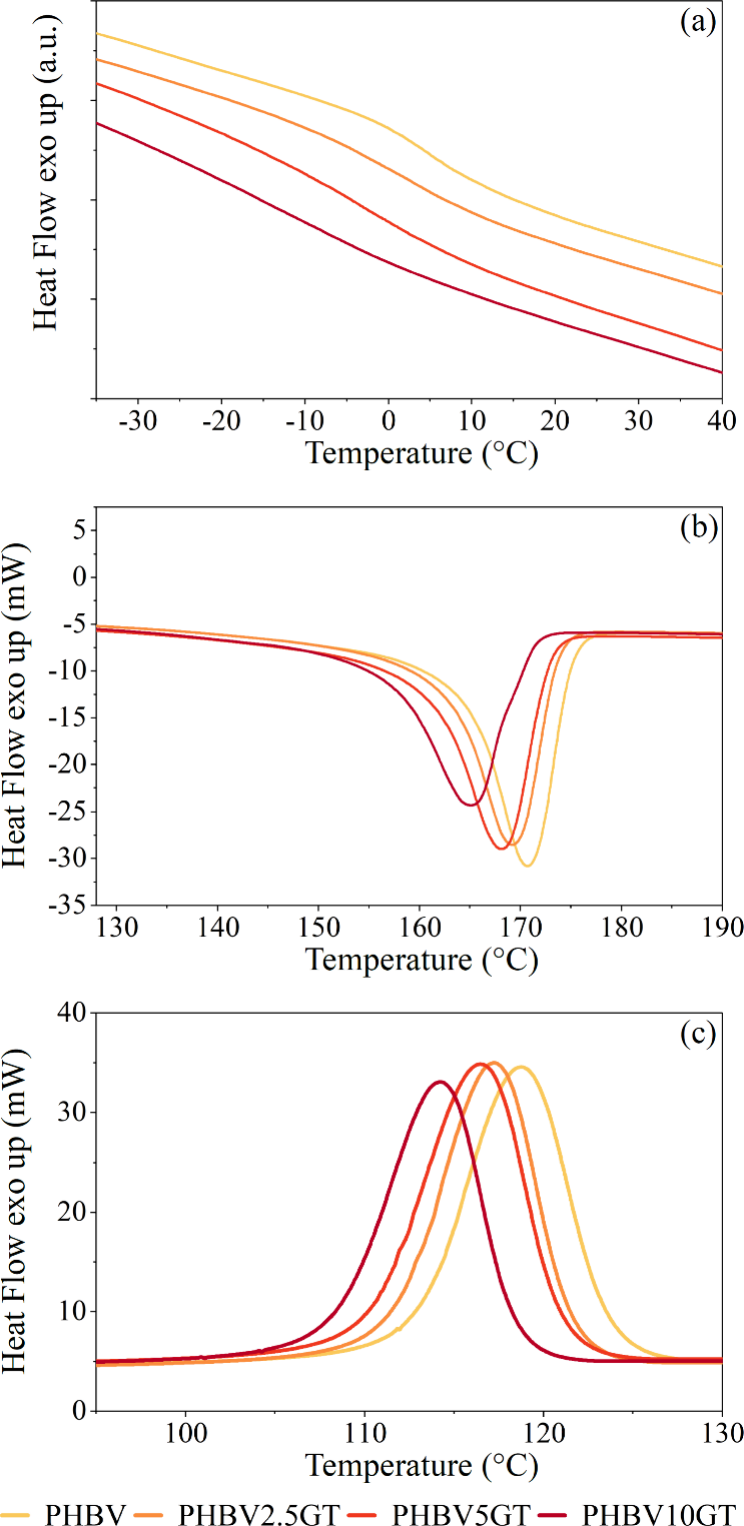
DSC thermograms of the PHBV/GT blends: (a) *T*_g_ inflection; (b) melting point endothermic peak; (c) crystallization point exothermic peak.

Furthermore, the presence of the plasticizer lowers both *T*_m_, defined as the temperature at which the crystalline regions transition from a solid to a liquid state, and *T*_c_, which is analogous to and opposite the aforementioned *T*_m_, since the crystallization transition occurs during cooling. The thermograms in Figure 2b and Figure 2c illustrate how the peaks of the melting and crystallization transitions, respectively, shift toward lower temperatures.

Simultaneously, both the melting (Figure 2b) and crystallization (Figure 2c) processes exhibit a decrease in intensity when the GT content is increased, which is typically related to a decrease in the transition enthalpy. The transition enthalpy of a polymer is defined as the energy that is provided (for the endothermic melting) or released (for the exothermic crystallization) to complete the phase transition and it is calculated as the area under the transition peak.^56^

Lower values of *T*_m_ and *T*_c_ might contribute to increased biodegradability, as has been found in literature.^57^ This is because the crystalline regions of plastics are less susceptible to biodegradation than the amorphous regions due to their more compact structure.^54^ The numerical values of thermal properties derived from the scans are reported in Table S3.

##### 3.1.2.2 TGA

The thermal stability of the material was not significantly affected by the plasticizer, as shown by the TGA results (Table 4). The onset of the degradation (*T*_onset_) was calculated at approximately 305 ± 2 °C by identifying the point of intersection between the plateau of the initial phase and the linear degradation phase. The temperature at which the maximum rate of degradation occurs, designated as the degradation temperature (*T*_d_), was derived from the inflection point on the TGA curve (Figure 3), which corresponds to the peak of the derivative.^58^ The mean *T*_d_ values were approximately 321 ± 1 °C across all compositions, indicating that varying plasticizer concentrations did not significantly affect the thermal degradation properties. This is particularly noteworthy, as the use of bioplasticizers often results in decreased thermal stability.^59,60^

**Table 4:** Thermal Degradation Parameters Obtained by TGA for the PHBV/GT Blends

**sample**	**degradation temperature (** *T*_d_ **) °C**	**onset temperature (** *T*_onset_ **) °C**	**residual mass (*m***_**r**_ **)** wt %
PHBV	322 ± 1	306 ± 1	0.6 ± 0.4
PHBV2.5GT	320 ± 2	304 ± 2	1.0 ± 0.1
PHBV5GT	321 ± 2	305 ± 2	0.9 ± 0.1
PHBV10GT	322 ± 1	306 ± 1	0.9 ± 0.2

**Figure 3:**
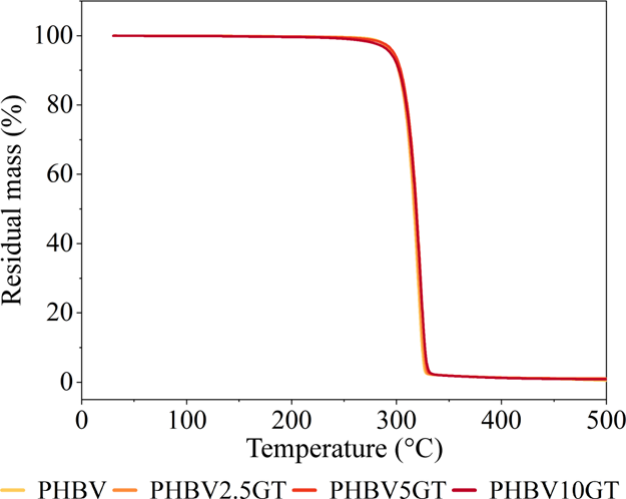
TGA thermograms obtained by analyzing the prepared PHBV/GT blends.

At the end of the heating ramp, more than 99% weight loss was achieved for all compounds, corresponding to approximately total degradation of the organic matter. In consideration of the processing temperatures employed with twin-screw extrusion, injection molding, and single-screw extrusion, the mass loss was determined to be less than 0.5%. This minimal loss is considered acceptable, likely attributable to the evaporation of residual moisture rather than any significant polymer degradation. As illustrated in Figure 3, the TGA curves for all the materials exhibited a single degradation event, indicating that the thermal degradation process occurs in a single step, further suggesting that the plasticizer is distributed homogeneously within the polymer matrix.^59^

These TGA resultsable support the claim that plasticizers have a negligible impact on the material’s overall thermal stability. This ensures consistency across the various formulations and guarantees that the materials can withstand the necessary processing conditions without being thermally affected by the temperatures involved in HTP.

### 3.2 Anaerobic Fermentation of PHBV with Varying Additive Concentrations

#### 3.2.1 Anaerobic Fermentation of Hydrothermally Pretreated PHBV

VFA production from hydrolyzed plastics was assessed at initial concentrations of 4 and 10 g SCOD/L (Figure 4a and Figure 4b). At the start of the fermentation, the medium’s composition was predominantly CA-3HB, indicating that the hydrolysis of PHBV10GT was successful during HTP. Trace amounts of *n*-butyrate (*n*-C_4_) and *n*-caproate (*n*-C_6_) were also initially present, likely introduced by the inoculum, as confirmed by control samples (Figure 4d). The unknown fraction of the initially supplied SCOD indicates the presence of unidentified molecules, such as oligomers and bioplasticizer portions, that could not be identified by GC. The presence of unknown compounds was further supported by the presence of unidentified peaks in the obtained chromatograms (Figure S3). HTP exhibited high hydrolysis efficiency, with 96% of the total COD (36.69 g COD/L, Calculation S1a) recovered as soluble SCOD (35.39 g SCOD/L, Section 2.2.2). This efficiency is comparable to that obtained with single PHBV under the same conditions.^61^ This represents a substantial enhancement over earlier research, which documented an efficiency level of merely 10% under more moderate conditions, characterized by lower temperatures, shorter duration, and reduced alkaline concentration.^40^

**Figure 4:**
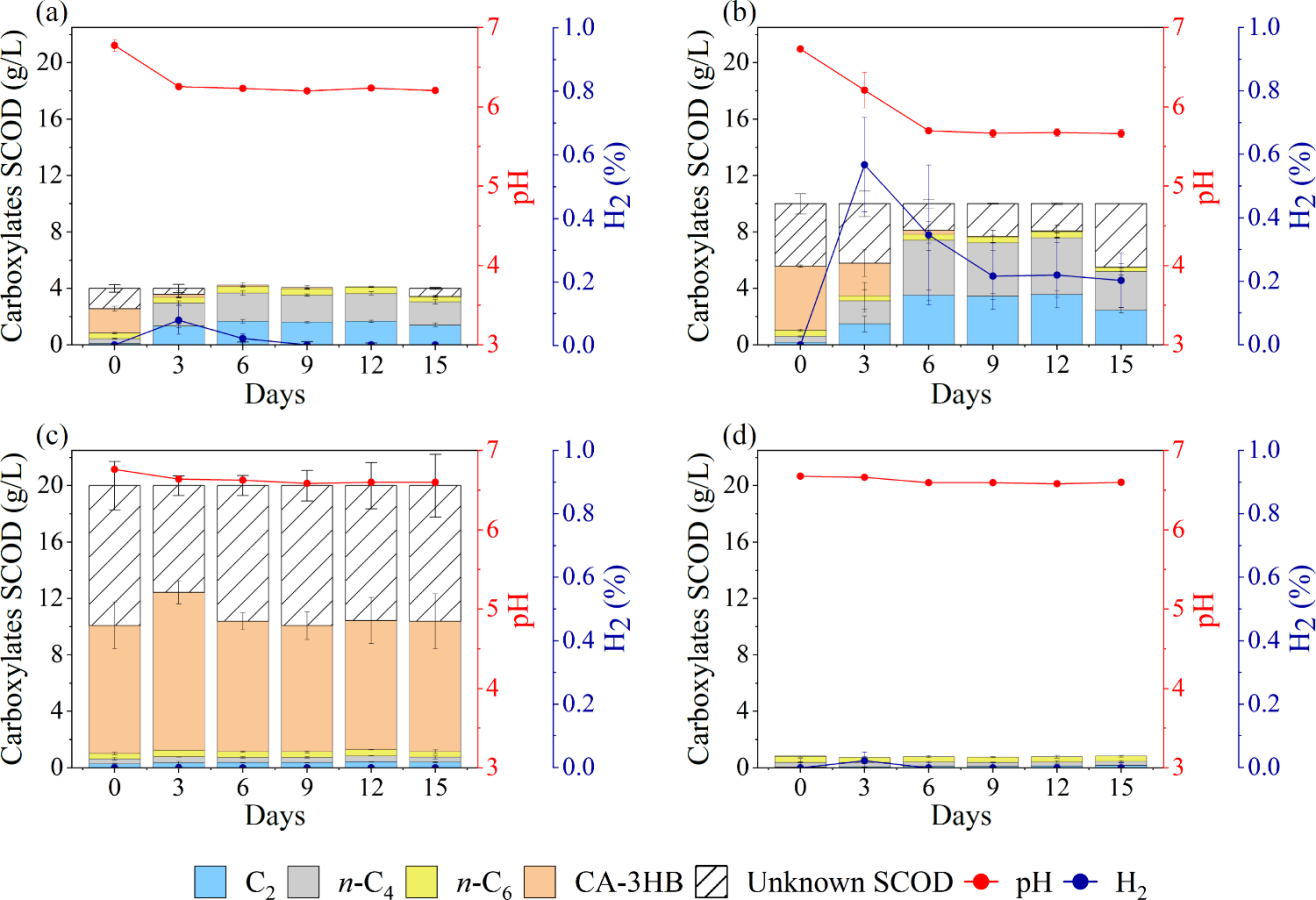
VFA production (stacked columns), pH (red line), and H_2_ (blue line) of (a) hydrolysates 4 g SCOD/L; (b) hydrolysates 10 g SCOD/L; (c) hydrolysates 20 g SCOD/L; and (d) control group with the inoculum alone. The CA-3HB concentrations were calculated by assuming only the SCOD values of crotonate. C_2_, *n*-C_4_, and *n*-C_6_ represent acetate, *n*-butyrate, and *n*-caproate, respectively. The error bars reported in the figures represent the standard deviation.

During fermentation, CA-3HB was fully consumed in both the 4 and 10 g SCOD/L experiments, with a corresponding increase in C_2_ and *n*-C_4_ and a decline in pH (Figure 4a,b). It is possible to hypothesize that different species of *Clostridium* present in the mixed inoculum are responsible for using CA-3HB as substrate.^61,62^ It is noteworthy that the production of VFA exhibited a correlation with pH trends. For both experiments, a decline in pH was accompanied by a reduction in CA-3HB and an increase in C_2_ and *n*-C_4_ levels. This VFA profile is consistent with previous findings on alkaline-pretreated PHA fermentation.^40^ Also, other recent work on mixed-culture crotonate fermentation, which used 100 mM (i.e., 15.2 g SCOD/L), showed a full conversion with the same kind of VFA products.^63^ While pH remained stable in the control and 20 g of SCOD/L samples, it dropped from 6.7 to 6.3 and 5.7 in the 4 and 10 g of SCOD/L fermentations, respectively, during the first 3–6 days. This pH decrement reached a plateau with the complete conversion of CA-3HB.^64^ Notably, in the 20 g SCOD/L experiment, CA-3HB was not converted to C_2_ and *n*-C_4_, indicating the potential microbial inhibition at this high hydrolysate concentration. This is consistent with previous reports on VFA concentration-dependent toxicity.^65^ These findings highlight that optimizing substrate concentration or setting up product extraction may support continuous conversion of PHBV hydrolysates (CA-3HB) to carboxylates.^66^

Minor amounts of hydrogen (H_2_) were detected in the headspace of the 4 and 10 g SCOD/L fermentations (Figure 4a and Figure 4b), likely as a byproduct of microbial metabolism of CA-3HB or *n*-butyrate oxidation to acetate.^63,67^ No H_2_ was detected in the control or 20 g of SCOD/L samples (Figure 4c). In anaerobic fermentation, H_2_ assumes a significant role as an intermediate during the acidogenic and acetogenic stages, primarily due to the activity of hydrogen-producing bacteria such as *Clostridium* spp.^68^ The presence of hydrogen serves as an indicator of active metabolic pathways and has the capacity to influence the subsequent formation of other products, including acetate or ethanol. Furthermore, hydrogen can be consumed by methanogenesis and redirected toward secondary products, depending on environmental conditions and microbial community interactions.^68^ The produced H_2_ amount decreased after an initial peak, suggesting that it could have been utilized in conjunction with CO_2_ for ethanol or acetate production.^69,70^ As expected, methane formation was not detected and, therefore, confirmed to be inhibited by the addition of BES.

The apparent complete microbial conversion of hydrolysate at 4 g SCOD/L demonstrates efficient fermentation and suggests potential conversion of both PHBV and maybe also some GT into VFA. The plasticizer GT is synthesized from esterification of levulinic acid and glycerol; its microbial conversion into carboxylate is plausible once the GT would break down into its building blocks during the hydrolysis and/or fermentation process.^28^ Enzymatic hydrolysis of GT showed already formation of levulinic acid and glycerol.^28^ Other studies show that levulinic acid can be partially converted into VFA, including acetate, by microbes. This process can even produce biogas, demonstrating its anaerobic digestibility.^71,72^ Additionally, glycerol has also been shown to be (partly) fermented into VFA by Clostridial species.^73,74^ Further experiments on sole GT digestion under methanogenic inhibited conditions could provide more insights into the plausible fermentability of the GT plasticizer into VFA.

#### 3.2.2 VFA Production and CA-3HB Accumulation during Anaerobic Fermentation of PHBV

The fermentation of PHBV/GT solid particles resulted in the production of primarily C_2_ and *n*-C_4_ (Figure 5). At the beginning of the experiment, only trace amounts of *n*-C_4_ and *n*-C_6_ were detected, with *n*-C_6_ originating from the inoculum, as confirmed by the control (Figure 5e). This is analogous to the findings in the HF experiments. As the experiment progressed, the production of C_2_ and *n*-C_4_ increased in all PHBV formulations until their concentrations reached a stable state from day 90 (Figure 5a–d). Conversely, CA-3HB was produced in minimal quantities for most of the experimental period, with a notable increase toward the conclusion of the experiment. Furthermore, it is noteworthy that the GC spectra (Figure S4) exhibited several unidentified peaks, in addition to the identified VFA, which could not be discerned using the current analytical method. It can be postulated that these peaks may represent intermediate compounds from plastic decomposition. This observations align with the findings that PHBV can be depolymerized into 3HB, crotonate, 3-HV, 2-pentenoate, and 3-pentenoate.^75^ Moreover, literature indicates that the biodegradation rate of PHBV is influenced by its monomeric composition, with higher 3-HV content accelerating the degradation through anaerobic fermentation,^76^ supporting the observation that structural variations in PHBV can significantly affect its breakdown behavior.

**Figure 5:**
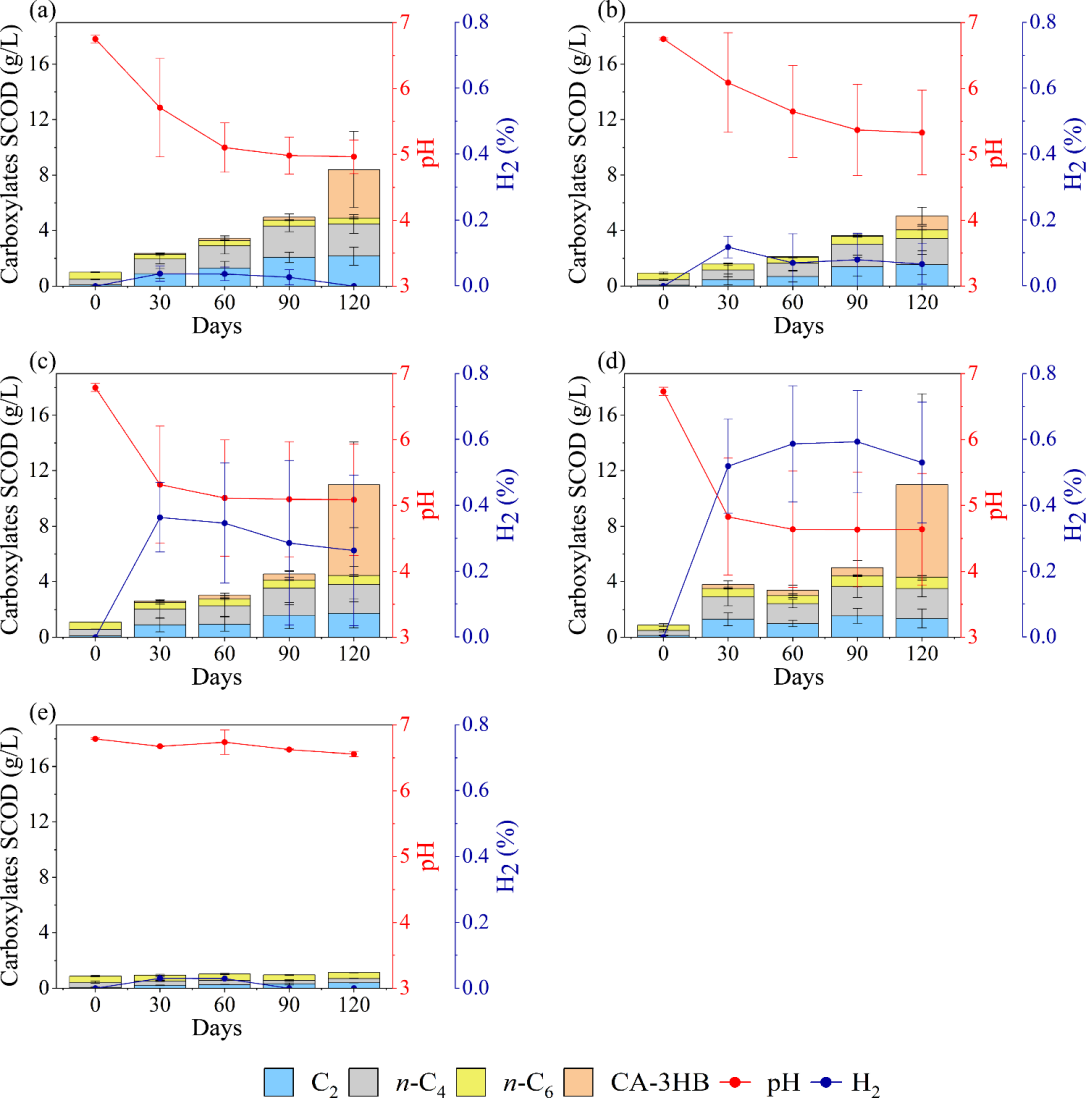
VFA production (stacked columns), pH (red line), and H_2_ (blue line) of (a) PHBV, (b) PHBV2.5GT, (c) PHBV5GT, (d) PHBV10GT, and (e) control. The CA-3HB concentrations were calculated by assuming only the SCOD values of crotonate. C_2_, *n*-C_4_, and *n*-C_6_ represent acetate, *n*-butyrate, and *n*-caproate, respectively. The error bars reported in the figures represent the standard deviation.

The fermentation was evidently initiated by the hydrolysis of plasticized PHBV into CA-3HB, followed by microbial conversion into carboxylates. Until day 90, hardly any CA-3HB accumulated, indicating that under these favorable conditions, hydrolysis and rapid microbial production of VFA occurred. In this period, the pH declined from 7 to around 5. Then, on day 120, CA-3HB clearly accumulated, and no additional VFA production was observed. As mentioned earlier, microbial toxicity for VFA is both concentration and pH-dependent,^77,78^ with increased toxicity observed as pH declines. This deducts that PHBV hydrolysis continued after day 90 but that microbial conversion was inhibited due to the nonfavorable conditions created by the low pH and presence of produced VFA and CA-3HB. This pH effect is in line with a recent work on mixed-culture crotonate fermentation, which showed microbial inhibition to occur at pH 5.9 (compared to pH 7.0) and no crotonate conversion at pH 5.5.

Not all supplied PHBV/GT particles were converted as visible by the eye (Figure S5) and shown by optical microscopic pictures (Figure S6). Referring to Figure 5d as a representative example, from the total COD supplied as PHBV10GT powder, equal to 85.61 g of COD_PHBV10GT_/L (Calculation S1b), approximately 5% was fermented into VFA within the first 90 days, 35 and 48% of which occurred as C_2_ (1.4 g/L) and *n*-C_4_ (1.2 g/L), respectively. At 120 days, 5% of the initial supplied COD was converted into VFA (1.3 g/L C_2_ and 1.2 g/L *n*-C_4_) while 8% (6.7 g/L) VFA occurred as CA-3HB. This evidence and the proposed fermentation mechanism highlight the importance of precise pH regulation for maintaining conditions that support sustained microbial conversion processes and further emphasize the necessity of promptly removing VFA upon reaching concentrations that exert inhibitory effects.

Notably, the composition of the gas phase demonstrated considerable variability in hydrogen production in response to alterations in the GT content. For the PHBV, PHBV2.5GT, and PHBV5GT formulations, hydrogen levels reached a maximum around day 30 and then stayed rather stable. The hydrogen concentrations in the PHBV10GT formulation reached the highest levels, but in all experiments, the concentration stayed below 1%, which is in line with the hydrothermally treated PHBV experiments.

Overall, the addition of the bioplasticizer GT did not significantly affect the biodegradation of PHBV, as indicated by consistent microbial metabolism and conversion to VFA, regardless of the GT concentration. The different characteristics of the formed bioplastics do therefore not hinder the microbial fermentation of the studied PHAs into VFA.

## 4 Conclusions

This study provides, for the first time, valuable insights into the anaerobic fermentation of plasticized PHBV with glycerol trilevulinate (GT). Compositions with different plasticizer contents showed reduced crystallinity, which may favor biodegradation and microbial conversion into VFA. The GT bioplasticizer was compatible with mixed-microbial culture metabolic activity. The findings underscore the ability of microorganisms, both with and without hydrolytic alkaline pretreatment of PHBV/GT blends, in generating VFA like acetate and *n*-butyrate, and several unidentified compounds. A low concentration of hydrolysate (4 g SCOD/L) facilitated a complete conversion of the hydrolysate into VFA; however, the highest concentration (20 g SCOD/L) hindered microbial activity, suggesting concentration-dependent toxicity to the present microbes. The study demonstrates that GT content in PHBV blends did not significantly influence the VFA production. Therefore, GT, in addition to being produced through a "green" synthesis process, is also compatible with PHBV fermenting microorganisms that produce VFA.

In further perspective, research could investigate the development of a controlled bioprocess that may utilize all supplied PHBV, as well the GT itself, and reveal if the unidentified products can also be used to predominantly produce short-chain length VFA. Such VFA has the potential to be used for various applications including the resynthesis of scl-PHAs polymers. Furthermore, a design of a bioplastic product, made with PHBV, GT, and other needed additives, could be aimed for and further studied on its properties including its microbial recyclability. Overall, the findings of this study indicate that PHBV/GT blends have the potential to be exploited as renewable biodegradable and microbially recyclable plastic materials.

## Supplementary Material



## Notes

(Table S1) Twin-screw extrusion conditions; (Table S2) composition of the stock solutions used to prepare the nutrient medium for the mixed-culture fermentation; (Figure S1) temperature and pressure profiles during the hydrothermal processing (HTP); (Figure S2) full DSC scans of PHBV and plasticized PHBV; (Table S3) thermal properties extrapolated from DSC thermograms; (Figure S3) chromatograms of the hydrolysate fermentation (HF) samples (a) before (day 0) and (b) after (day 15) the fermentation; (Calculation S1) COD calculations for (a) PHBV10GT supplied for the hydrothermal processing (HTP) and (b) PHBV10GT supplied for the solid fermentation, as representative examples; (Figure S4) chromatograms of the solid fermentation (SF) samples at day 120; (Figure S5) PHBV/GT solid particles not converted at the end of the fermentation process (day 120) as visible by the eye; (Figure S6) optical microscopy of the powder materials before (day 0, top line) and after (day 120, bottom line) the anaerobic fermentation (SF experiment) (PDF)

## Abbreviations:

3HB: 3- hydroxybutyrate
3-HV: 3- hydroxyvalerate
BES: sodium 2-bromoethanesulfanoate
C_2_: acetate
CA: crotonic acid
COD: chemical oxygen demand
Δ*H*_c_: crystallization enthalpy
Δ*H*_m_: melting enthalpy
DSC: differential scanning calorimetry
GC: gas chromatography
GT: glycerol trilevulinate
HF: hydrolysate fermentation
HTP: hydrothermal pretreatment
*m*_r_: residual mass
*n*-C_4_: *n*-butyrate
*n*-C_6_: *n*-caproate
PHAs: poly­(hydroxyalkanoates)
PHB: poly­(3-hydroxybutyrate)
PHBV: poly­(3-hydroxybutyrate*-co-*3-hydroxyvalerate)
SCOD: soluble chemical oxygen demand
SEM: scanning electron microscopy
SF: solids fermentation
*T*_c_: crystallization temperature
*T*_d_: thermal degradation temperature
*T*_g_: glass transition temperature
TGA: thermogravimetric analysis
*T*_m_: melting temperature
*T*_onset_: onset temperature
VFA: volatile fatty acids
χ_c_: degree of crystallization
