# Supplementary material for: Anaerobic Fermentation of Poly(3-hydroxybutyrate-co-3-hydroxyvalerate) Plasticized with Glycerol Trilevulinate into Volatile Fatty Acids
Source: ACS Omega. 2026 Mar 31;11(14):22308–19. doi: 10.1021/acsomega.6c00869 (PMC13084440; doi:10.1021/acsomega.6c00869)
Supplement: Supplementary file 1 [file ao6c00869_si_001.pdf]

Supporting Information

Anaerobic Fermentation of Poly(3-Hydroxybutyrate-co-3-hydroxyvalerate) plasticized with Glycerol Trilevulinate into Volatile Fatty Acids

Elena Togliatti [a,b,1], Yong Jin [c,1], Luca Lenzi [b,d], Davide Morselli [b,d], Micaela Degli Esposti [b,d], Paola Fabbri [b,d], Daniel Milanese [a,b], David P. B. T. B. Strik [c,*], Corrado Sciancalepore [a,b,**]

a. Department of Systems and Industrial Technologies Engineering, Università di Parma, Parco Area delle Scienze 181/A, 43124 Parma, Italy

b. INSTM, National Interuniversity Consortium of Materials Science and Technology, Via G. Giusti 9, 50121 Firenze, Italia

c. Environmental Technology, Wageningen University & Research, 6708 WG Wageningen, The Netherlands

d. Department of Civil, Chemical, Environmental and Materials Engineering, Università di Bologna, Via Terracini 28, 40131 Bologna, Italy

1 Authors contributed equally

* Corresponding author at: Environmental Technology, Wageningen University & Research, 6708 WG Wageningen, The Netherlands

** Corresponding author at: Department of Engineering for Systems and Industrial Technologies, Università di Parma, Parco Area delle Scienze 181/A, Parma 43124, Italy.

Table S1. Twin screw extrusion conditions.

Head	Zone 6	Zone 5	Zone 4	Zone 3	Zone 2	Zone 1	Hopper	Screw speed
150 ± 1 °C	158 ± 1 °C	160 ± 1 °C	162 ± 1 °C	162 ± 1 °C	155 ± 1 °C	150 ± 1 °C	40 ± 1 °C	30 rpm

Table S2. Composition of the stock solutions used to prepare the nutrient medium for the mixed culture fermentation.

	Compound	Concentration (g/L)	Dilution factor
Stock solution I – Minerals	$NH_4H_2PO_4$	180	50x
	$MgCl_2 \cdot 6H_2O$	16.5	
	$MgSO_4 \cdot 7H_2O$	10	
Stock solution II – Minerals	$CaCl_2 \cdot 2H_2O$	10	50x
	KCl	7.5	
Trace metals	$FeCl_2 \cdot 4H_2O$	30	2000x
	$MnCl_2 \cdot 4H_2O$	0.6	
	H_3BO_4	6.0	
	$CoCl_2 \cdot 6H_2O$	4.0	
	$CuCl_2 \cdot 2H_2O$	0.2	
	$NiCl_2 \cdot 6H2O$	0.4	
	$ZnSO_4 \cdot 7H_2O$	2.0	
	$Na_2MoO_4 \cdot 4H_2O$	0.6	
	Na_2SeO_3	0.2	
	EDTA	12.4	
Vitamins	Biotin	0.106	1000x
	Folic acid	0.005	
	Pyridoxal-HCl	0.0025	
	Lipoic acid	0.015	
	Riboflavin	0.0125	
	Thiamin-HCl	0.413	
	Ca-D-panthotene	0.0125	
	Cyanocobalamin	0.0125	
	P-aminobenzoic acid	0.0125	
	Nicotinic acid	0.0125	
Supplements	Yeast extract	0.1	
	KOH	4M	

Notes: After all trace metals were added, the pH in the trace metals stock solution was adjusted to 1.0 by adding 37% HCl.

Figure S1. Temperature and pressure profiles during the hydrothermal processing (HTP).

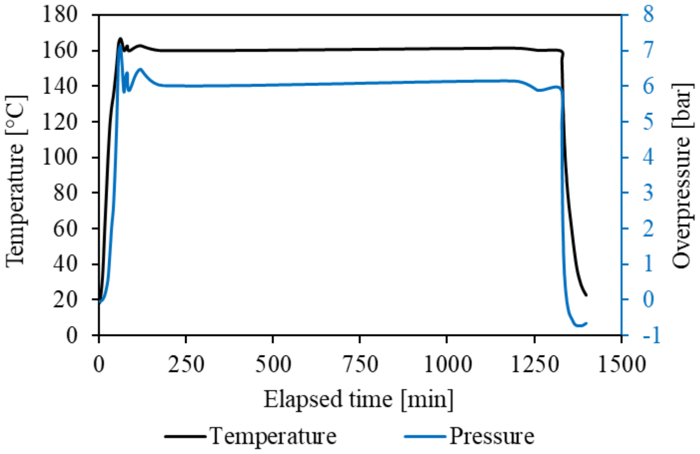

Figure S2. Full DSC scans of PHBV and plasticized PHBV

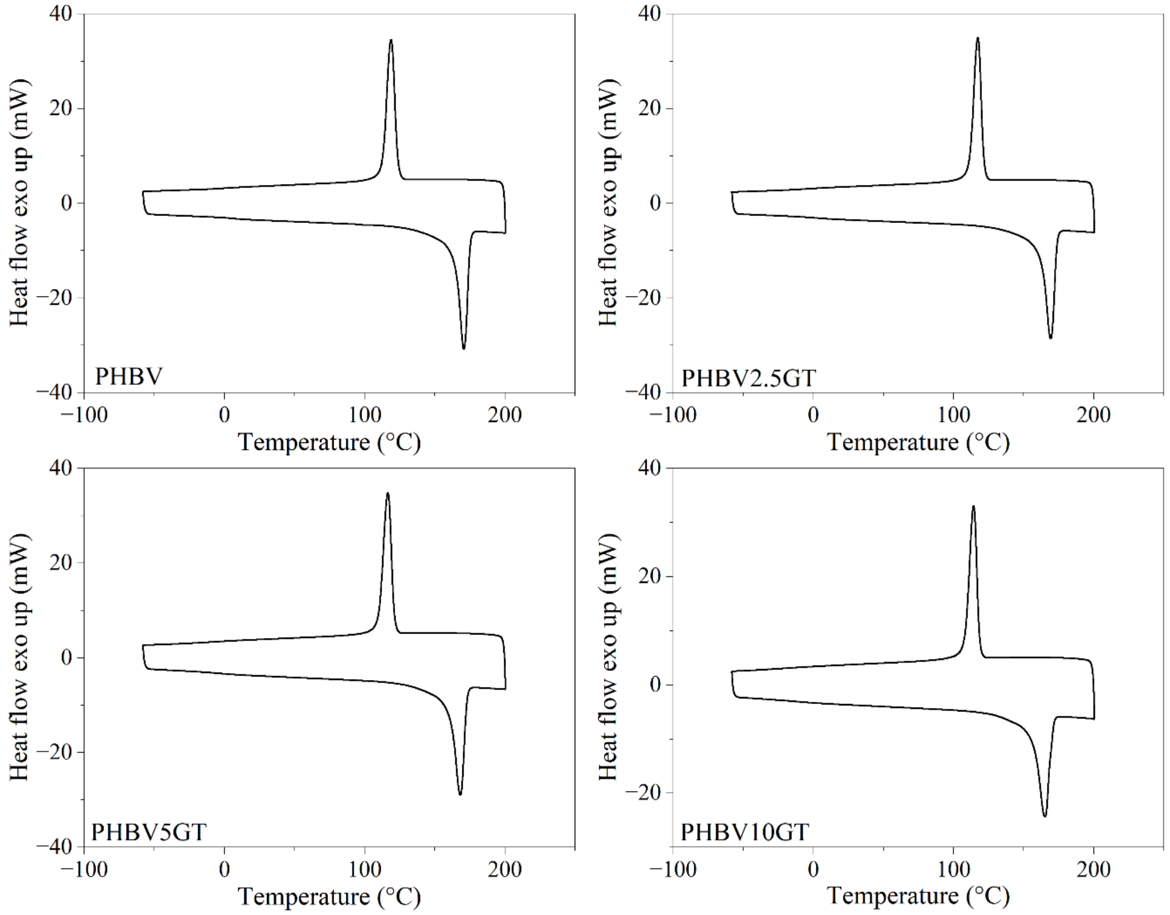

Table S3. Thermal properties extrapolated from DSC thermograms.

	T_g (°C)	T_m (°C)	ΔH_m (J/g)	T_c (°C)	ΔH_c (J/g)	χ_c (%)
PHBV	4.8 ± 0.7	170.7 ± 0.2	108.8 ± 2.5	118.7 ± 0.1	99.3 ± 2.7	75 ± 2
PHBV2.5GT	3.3 ± 0.7	169.2 ± 0.1	103.0 ± 3.5	117.2 ± 0.2	93.9 ± 2.9	72 ± 2
PHBV5GT	-1.6 ± 3.1	168.1 ± 0.2	105.9 ± 3.9	116.5 ± 0.1	96.2 ± 3.2	76 ± 3
PHBV10GT	-9.1 ± 1.1	165.1 ± 0.1	94.2 ± 5.0	114.2 ± 0.1	84.8 ± 4.7	72 ± 4

Figure S3. Chromatograms of the hydrolysate fermentation (HF) samples (a) before (day 0) and (b) after (day 15) the fermentation. Unidentified peaks are highlighted with red arrows.

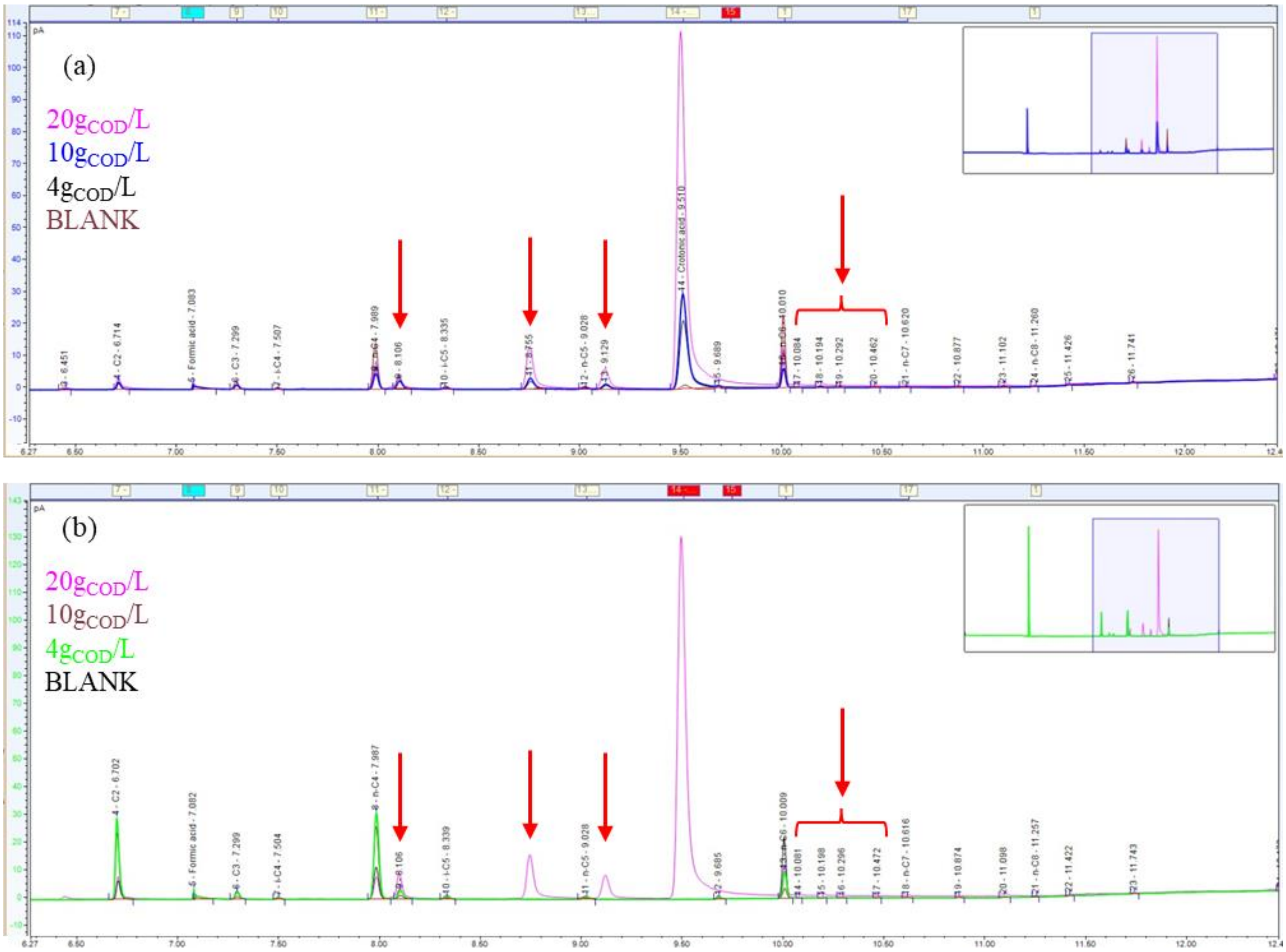

Calculation S1. COD calculations for a) PHBV10GT supplied for the hydrothermal processing (HTP) and b) PHBV10GT supplied for the solids fermentation, as representative example.

The PHBV utilized in this work has a composition of 97% PHB and 3% PHV comonomers.

COD_{PHB} = 1.6744 g COD /g_{PHB}

COD_{PHV} = 1.92 g COD /g_{PHV}

COD_{PHBV} = 0.97 · COD_{PHB} + 0.03 · COD_{PHV} = 0.97 · 1.6744 g COD/g_{PHB} + 0.03 · 1.92 g COD/g_{PHV} = 1.6816 g COD/g_{PHBV}

COD_{GT} = 1.9877 gCOD/g_{GT}

a) The supplied material for HTP was 15g of plasticized PHBV10GT: 13.5g are of PHBV and 1.5g are of glycerol trilevulinate bio-plasticizer.
   $COD_{PHBV10GT}$ = 13.5g · 1.6816 g COD/g_{PHBV} + 1.5g · 1.9877 g COD/g_{GT} = 25.6815 g $COD_{PHBV10GT}$
   The concentration of the COD in the reactor volume is: 25.6815 g $COD_{PHBV10GT}$ / 0.7 L = 36.6879 $COD_{PHBV10GT}$/L.

b) The supplied material for SF was 2.5g of plasticized PHBV. In the case of PHBV10GT: 2.25g are of PHBV and 0.25g are of glycerol trilevulinate bio-plasticizer.
   $COD_{PHBV10GT}$ = 2.25g · 1.6816 g COD/g_{PHBV} + 0.25g · 1.9877 g COD/g_{GT} = 4.2805 g $COD_{PHBV10GT}$
   The concentration of the COD in the reactor volume is: 4.2805 g $COD_{PHBV10GT}$ / 0.05 L = 85.61 g $COD_{PHBV10GT}$/L.

Figure S4. Chromatograms of the solid fermentation (SF) samples at day 120. Unidentified peaks are highlighted with red arrows.

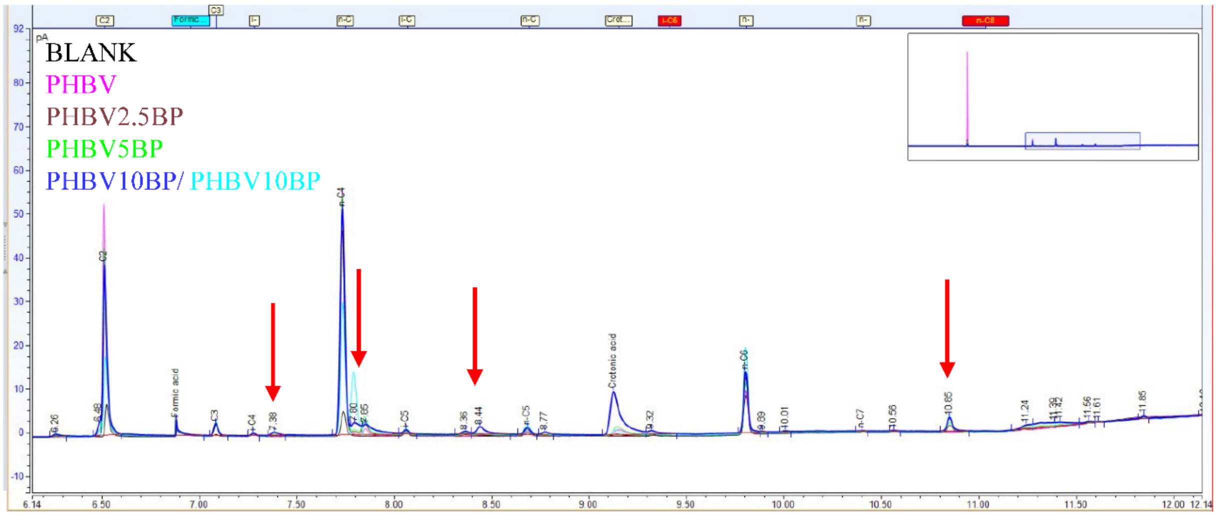

Figure S5. PHBV/GT solid particles not converted at the end of the fermentation process (day 120) as visible by eye.

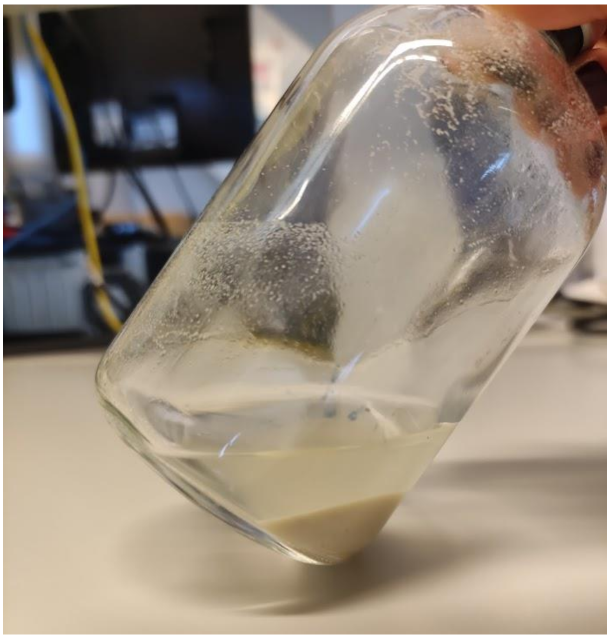

Figure S6. Optical microscopy of the powder materials before (day 0, top line) and after (day 120, bottom line) the anaerobic fermentation (SF experiment).

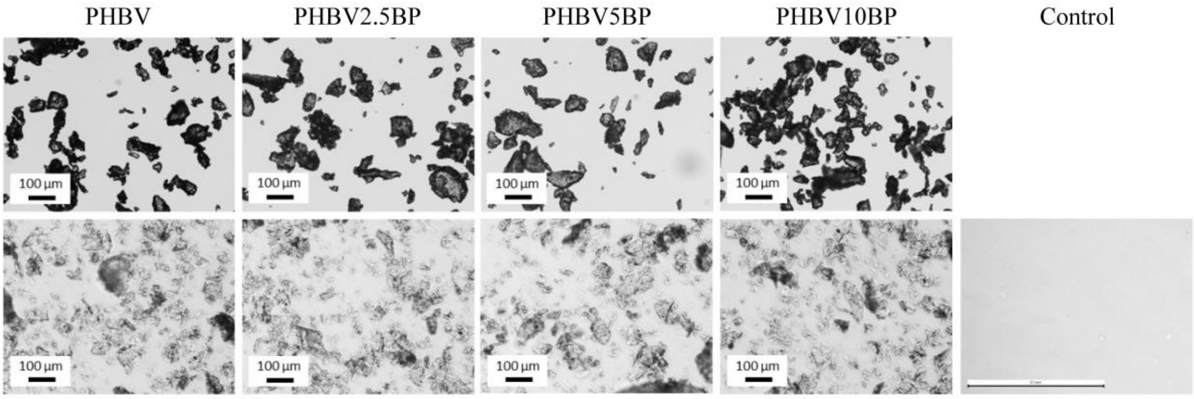

The images displayed in the top row illustrate the dry, milled materials in their original state before their introduction in to the reactors. The bottom row depicts particles immersed in the reaction liquid at the end of the experiment. The alteration in opacity may be attributed to the different imaging conditions, with the top row images captured in the absence of a liquid medium, while the bottom row images depict particles within the fermentation liquid.
